# TET2 Mutations in Ph-Negative Myeloproliferative Neoplasms: Identification of Three Novel Mutations and Relationship with Clinical and Laboratory Findings

**DOI:** 10.1155/2013/929840

**Published:** 2013-05-25

**Authors:** Andrea Patriarca, Donatella Colaizzo, Gianluca Tiscia, Raffaele Spadano, Silvia Di Zacomo, Antonio Spadano, Ida Villanova, Maurizio Margaglione, Elvira Grandone, Alfredo Dragani

**Affiliations:** ^1^Bleeding and Thrombotic Disorders Unit, Department of Hematology, Ospedale Civile dello “Spirito Santo”, 65124 Pescara, Italy; ^2^Atherosclerosis and Thrombosis Unit, IRCCS “Casa Sollievo della Sofferenza”, 71013 San Giovanni Rotondo, Italy; ^3^Laboratory of Molecular Biology, Department of Hematology, Ospedale Civile dello “Spirito Santo”, 65124 Pescara, Italy; ^4^Department of Medical Genetics, Foggia University, 71100 Foggia, Italy

## Abstract

High-throughput DNA sequence analysis was used to screen for TET2 mutations in peripheral blood derived DNA from 97 patients with BCR-ABL-negative myeloproliferative neoplasms (MPNs). Overall six mutations in the coding region of the gene were identified in 7 patients with an overall mutational frequency of 7.2%. In polycythemia vera patients (*n* = 25) 2 mutations were identified (8%), and in those with essential thrombocythemia (*n* = 55) 2 mutations (3.6%); in those with unclassifiable MPN (*n* = 8) 3 mutations (37.5%). No primary myelofibrosis patients (*n* = 6) harboured TET2 mutations. Three unreported mutations were identified (p.P177fs, p.C1298del, and p.P411del), the first two in patients with unclassifiable MPN, the last in a patient with essential thrombocythemia. On multivariate analysis the diagnosis of an unclassifiable MPN was significantly related to the presence of TET2 mutations (*P* = 0.02; OR: 2.81; 95% CI 1.11–7.06). We conclude that TET2 mutations occur in both JAK2 V617F-positive and -negative MPNs and are more frequent in MPN-U patients. This could represent the biological link between the different classes of myeloid malignancies.

## 1. Introduction

Philadelphia-negative myeloproliferative neoplasms (MPNs) are a spectrum of clonal disorders of the hematopoietic system characterized by overproduction of mature blood elements, a trend to thrombotic and/or hemorrhagic complications with variable rates of transformation to secondary myelofibrosis and acute leukemia [1]. The presence of JAK2 or MPL mutations represents major diagnostic criteria in the WHO classification of classic BCR-ABL-negative MPNs. However, a variable percentage of patients lack both molecular markers. The molecular basis of JAK2- and MPL-negative MPN remains largely unexplained. Recently, new molecular markers have been described in a vast array of myeloid cancers [1–3]. In particular, alterations in the TET2 gene, a putative tumor suppressor gene located at chromosomal region 4q24, have been identified in 7–13% of MPN patients, in 19–26% myelodysplastic syndromes (MDSs), in 12–24% of acute myeloid leukemia (AML), in 20–40% of chronic myelomonocytic leukemia (CMML), and in 29% of systemic mastocytosis [2–9]. Moreover, at the best of our knowledge, no significant correlation was observed between the TET2 mutation status and both the clinical-laboratory phenotype and the risk of secondary clonal evolution in MPNs [6]. Aims of our study were (1) to investigate the presence of TET2 mutations in MPN patients with or without the JAK2 V617F mutation and (2) to establish a possible relationship between clinical and laboratory findings in the context of polycythemia vera (PV), essential thrombocytemia (ET), primary myelophibrosis (PMF) and myeloproliferative neoplasms unclassifiable (MPNs-U).

## 2. Materials and Methods

### 2.1. Patients

After approval by the institutional review board, we selected from our database 98 MPNs adult patients (26 PV, 55 ET, 6 prefibrotic and 3 fibrotic PMF, and 8 MPN-U), diagnosed according to WHO 2008 diagnostic criteria [10]. At the time of enrolment, 8 patients showed clinical and laboratory pictures compatible with a secondary evolution as follow: 3 post-ET PMF, 2 post-PV PMF, 1 PV in accelerated phase, 1 PMF evolved in CMMoL, and 1 MPN-U in secondary fibrosis. All parameters used for statistical analysis, except for those addressing prognosis (survival, leukemic or fibrotic transformation), were those obtained at the time of diagnosis and before any therapeutic intervention. A complete data set of all the major clinical characteristics is available online (see supplementary material available online at http://dx.doi.org/10.1155/2013/929840).

### 2.2. Molecular Biology

Mutation screening for JAK2 V617F was performed on granulocyte DNA from peripheral blood (PD) samples at Laboratory of our department, according to the procedure previously described [11]. High-throughput DNA sequence analysis was used to screen for TET2 mutations in PB-derived DNA at Atherosclerosis and Thrombosis Unit, IRCCS Casa Sollievo della Sofferenza, San Giovanni Rotondo, Italy. Briefly, all the exons, introns, and 5′UTR of the gene were amplified using forward and reverse PCR primer designed according to the DNA sequence reported in the literature (reference sequence NC 000004). PCR primers were designed to amplify and sequence <500 bp amplicons, and overlapping PCR amplicons were designed for all exons >400 bp to ensure complete coverage. For each PCR reaction, 50 ng of genomic DNA was used. All the PCR products were sequenced on ABI PRISM 3100 Genetic Analyzer sequencer (PE Biosystems, Foster City, CA, USA). All the frameshift and nonsense mutations were scored as pathological mutation. Point mutations were excluded if they were synonymous mutations or included in SNP database (http://www.ncbi.nlm.nih.gov/projects/SNP/). DNA was available from 97 patients for TET2 sequencing. In one case (suffering from PV complicated by portal vein thrombosis), we were unable to extract DNA due to sample unsuitability. 

### 2.3. Statistical Analyses

Statistical analyses were performed using MedCalc 11.6.1 (1993–2011, Mariakerke, Belgium, Europe). All *P* values were two tailored, and the level of significance was set at the level of *P* < 0.05. Continuous variables were shown as median and range. Categorical variables were described as frequency and percentage. Comparison between categorical variables was performed by Chi-squared statistics. Comparison between categorical and continuous variables was performed by Mann-Whitney *U* test or Kruskal-Wallis test, where appropriate. The association between the variables selected from univariate analysis and the TET2 mutational status was explored using logistic regression, using as confounding factors age, gender, JAK2 V617F mutation frequency, monocyte, Plt and WBC count, Hb, cellularity on bone marrow trephine biopsy, and cytoreductive treatment. 

## 3. Results

The diagnostic and clinical characteristics of the whole cohort sorted by diagnosis are summarized in Table 1.

In addition to the expected differences in full blood count, frequency of splenomegaly, JAK2 V617F allele burden, bone marrow cellularity, depending on specific MPN subtype, we found that the PV patients were significantly older than the other patients (*P* = 0.04), and ET patients were more often females (*P* = 0.04). 

Overall, we found 6 mutations all in the coding sequence of the gene in 7 patients (Table 2) with an overall prevalence of 7.2%. No intron boundaries mutations were found. No significant difference was observed between patients with/without TET2 mutations as far as the JAK2 V617F frequency is concerned (Table 3). The same conclusion was reached when we correlated the JAK2 V617F allele burden with TET2 mutation status both in the whole cohort and after the exclusion of PV patients.

Interestingly, 3 patients harboured novel TET2 mutations: 2 in the exon 3 (P39 and P98) and 1 in the exon 7 (P42) mutations. The first (p.P177fs) is a frameshift mutation caused by the deletion of cDNA cytosine 530, the second (p.P411del) is a proline deletion due to the c.1232-1234delCCT. The third novel mutation (p.C1298del) in the exon 7 is due to the deletion of c.3890_3892delGAT.

The other three mutations identified are reported in Table 2 and were previously described. A significantly higher prevalence of TET2 mutations was recorded in the MPN-U (3/8, 37.5%) with an OR of 12.72 (95% CI: 2.21–73.2; *P* = 0.005). A Plt count below the whole group median count (615 × 10^6^/L) and to a minor frequency of spontaneous MK colony growth was observed in patients carrying TET2 mutations (*P* = 0.02 (OR: 8.58; 95% CI 1.1–74.4) and *P* = 0.01 (OR: 10.3; 95% CI: 1.19–89.87), resp.). On multivariate analysis, the presence of TET2 mutation was significantly and independently associated only with MPN-U diagnosis (*P* = 0.02 (OR: 2.81; 95% CI 1.11–7.06)). 

Moreover a monocytic proliferation not reaching the minimum criteria for a diagnosis of CMML (<10%) was recoded by trephine biopsies from all the 3 TET2 mutated MPN-U patients without a significant peripheral monocytosis at diagnosis as showed in Table 3. However the same evaluation was not available for the other 4 TET2 mutated patients and for the TET2-wt controls. 

Twenty-nine major thrombotic events were recorded at diagnosis in the whole cohort (Table 1): 3 of them (10.3%) occurred in patients carrying TET2 mutation (Table 3). The first event was an acute myocardial infarction recorded in a 43-year-old patient diagnosed with ET; the other 2 patients were both affected by PV and showed a deep vein thrombosis at diagnosis. As far as the 25 thrombotic events in patients without TET2, 3 portal vein thrombosis, 1 Budd-Chiari syndrome, 4 strokes, 2 deep vein thrombosis, and 13 acute myocardial infarctions were recorded (Table 4). As stated before in 1 case of myocardial infarction information about TET2 mutation was not available (Table 3).

Four bleeding episodes (2 posttraumatic and 2 spontaneous) were recorded during followup, all in patients without TET2 mutation. The posttraumatic haemorrhages were as follows: 1 episode posthysterectomy in IMF patient and 1 in ET patient following Caesarean section due to dystocic labour. Both patients had received low-molecular-weight heparin as standard prophylaxis for deep vein thrombosis at the time of haemorrhagic complication. Moreover, only in ET patient a Plt count approaching 1.000 × 10^9^/L was recorded. 

Two patients showed a spontaneous haemorrhage: the first was a PV patient with subdural haemorrhage and the latter was an ET patient. In both cases a Plt count above 1.000 × 10^9^/L was recorded without significant other coagulation abnormalities.

Eight patients (8.2%) showed clinical-laboratory findings compatible with secondary evolution (3 post-ET MF, 2 post-PV MF one of which underwent allogenic stem cell transplantation, 1 post-PV accelerated phase, 1 evolution into CMMoL, 1 MPN-U evolving into secondary fibrosis) after a median followup of 68.2 months (range 32.4–146.2 months). In this subgroup, only one PV patient (P21) evolving into accelerated phase carried one of the TET2 mutations. Moreover, in the whole cohort we registered only one death due to postsurgical sepsis (in 1 patient suffering from PV). 

## 4. Discussion 

The TET2 mutational frequency observed in this study (7.2%) is similar to that reported by other authors [3, 6, 12] in both in sporadic and in familiar MPNs. Moreover, our data confirm data by Tefferi et al. [6] about the lower frequency of TET2 mutations in patients without JAK2 V617F.

Interestingly, we find three novel mutations in the coding sequence of TET2, two located within the exon 3 and one on exon 7. The first mutation is a deletion of a cytosine 530 leading to a frameshift and possibly to a premature stop codon resulting in a nonfunctional protein. The second one is a tribase deletion from 1232 to 1234 resulting in the p.P411del. The last one is a GAT deletion from 3890 to 3892 on cDNA. The exact effect of these last two deletions is unknown, but they likely result in an abnormal protein folding. The other TET2 mutations previously reported by other authors, with the exception of p.Q652fs, have never been reported in MPNs patients [12–20]. p.Q652fs was previously described [12, 13] in a familiar ET with double heterozygous TET2 status evolving after a brief observation into secondary AML and in a type I CMML. p.R1572W was for the first time identified by Kosmider et al. in a type I CMML [14]; p.V1718L was described by several groups [15–20] mainly in MDS and MPN/MDS.

In agreement with Tefferi et al. [8], we find a significant correlation between TET2 mutation and an MPN-U diagnosis. Even if the small sample size does not allow firm conclusion, we can argue that TET2 is the biological substrate linking MPNs, MPN/MDS, MDS, and AML. This hypothesis is supported by the absence of a significant difference in clinical and laboratory features between TET2 mutated and wt patients in MPNs and by the increasing frequency of the mutation passing from through the different classes of diseases. No other significant differences were found between clinical and laboratory data at diagnosis sampling the cohort by TET2 mutation and diagnosis. More in detail, our data do not confirm the correlation with the age in MPNs, underlined by Tefferi et al. [6]. This is probably due to the lower prevalence (8%) of the mutation in PV group, which in our sample is older than the other groups.

Moreover, according to data previously published by Nguyen-Khac in transformed MPNs, the presence of any TET2 mutation does not relate to other genetic aberrations detected with karyotype and FISH examination on chromosome from bone marrow hematopoietic precursors [13]. 

Due to the low number of patients progressing to secondary fibrosis or secondary hematopoietic malignancy, we were not able to evaluate OS and EFS, and thus if TET2 mutations are a detrimental or a favourable factor in secondary clonal evolution in MPNs.

However, with all the limitations due to the small sample size and to the lack of a specific assay in vitro assay, we can speculate, according to the data from Ko et al. [17], that the presence of the TET2 mutation can induce a second clone with a prominent monocytic differentiation. This second clone can affect the morphology of bone marrow leading to morphological diagnosis of MPN-U. These findings could also explain the occurrence of the clonal evolution of MPN to a MPN/MDS phenotype. 

Our data confirm and extend those previously published and allow us to speculate that TET2 mutations could represent the biological link between different classes of myeloid malignancies. 

## Supplementary Material

Clinical and Laboratory data of 97 MPN's patients at diagnosis: the only differences we found in our cohort were those related to the specific subtype of MPN. No other significant differences were observed. However we found a significant prevalence of the TET2 mutation in MPN-U sub-type. Of interest 3/7 of the TET2 mutation observed were new mutation not described elsewhere

## Figures and Tables

**Table 1:** Clinical and laboratory characteristic of 98 MPNs at diagnosis according to diagnosis.

	IMF	MPN-U	PV	TE	*P* value
Number of patients	9	8	26	55	na
M/F	8/1	3/5	17/9	25/30	0.04
Median age, years (range)	68 (54–82)	69 (43–67)	72 (30–85)	63 (25–89)	0.04
Follow-up days, median (range)	885.5 (397–2130)	1314 (350–2707)	1358.5 (128–5871)	2137 (179–4708)	0.1
Hb, g/100 mL (range)	15.1 (12.1–17.4)	16.1 (12.5–19.1)	18.0 (14.5–21.5)	14.9 (11.1–18.1)	0.0001
WBC × 10^9^/L (range)	7.43 (5.52–13.82)	7.73 (5.4–15.53)	10.98 (1.7–19.6)	8.3 (3.18–16.71)	0.05
Monocytes × 10^9^/L (range)	0.48 (0.08–0,89)	0.4 (0.3–0.82)	0.45 (0.02–1.55)	0.45 (0.11–0.78)	0.8
Plt × 10^9^/L (range)	615 (429–1094)	629 (181–1006)	481 (134–1745)	684 (443–1750)	0.0001
JAK2 V617F *n* (%)	6/9 (66.6)	6/8 (75)	26/26 (100)	35/55 (63.6)	0.0001
JAK2 V617F burden median % (range)	40 (5–64)	17 (4–40)	60 (24–90)	14 (5–59)	0.0001
EEC *n* (%)	6/9 (66.6)	6/8 (75)	23/26 (88.5)	41/55 (74.5)	0.1
CGU-MK *n* (%)	3/9 (33.3)	4/8 (37.5)	16/26 (61.5)	36/55 (65.5)	0.1
CD34 + SP × 10^9^/L (range)	0.051 (0.013–0.25)	0.021 (0.0046–0.051)	0.040 (0.0035–0.5)	0.024 (0.0015–0.61)	0.07
CD34 + SP (%) (range)	0.05 (0.035–0.45)	0.03 (0.01–0.09)	0.04 (0.01–0.56)	0.03 (0.0015–0.27)	0.07
Bone marrow cellularity % (range)	60 (30–90)	40 (20–80)	80 (60–95)	50 (20–98)	0.0001
Single cytogenetic abnormality (*n*)	1/9	2/8	2/26	0/54	**0.3**
Complex karyotype (*n*)	0/9	0/8	0/26	1/54	
Trisomy (*n*)	0/9	0/8	1/26	0/54	
Palpable splenomegaly (*n*)	6/9	3/8	16/26	15/55	0.7
Splenic square cm^2^ (range)	50 (26–102)	43,5 (37–65)	63 (25–200)	40 (28–105)	0.04
Arterial thromboses (*n*)	5/9	0/8	6/26	12/55	0.6
Venous thromboses (*n*)	1/9	0/8	6/26	3/55	
Spontaneous haemorrhage (*n*)	0/9	0/8	1/26	1/55	0.7
Posttraumatic haemorrhage (*n*)	1/9	0/9	0/26	1/55	
Cytoreductive treatment (*n*)	7/9	7/8	26/26	42/55	0.06

**Table 2:** TET2 mutations in MPN patients.

	Sequence location	Effect	cDNA	Patient ID (diagnosis)
p.P177fs	Exon 3	Frameshift	c.530delC	P39 (MPN-U)
p.P411del	Exon 3	Deletion	c.1232_1234delCCT	P98 (TE)
p.C1298del	Exon 7	Deletion	c.3890_3892delGAT	P42 (MPN-U)
p.Q652fs*	Exon 3	Frameshift	c.1954delC	P65 (MPN-U)
p.R1572W*	Exon 11	Missense	c.4714C>T	P23 (PV)
p.V1718L*	Exon 11	Missense	c.5152G>T	P21, P25 (TE, PV)

*Novel mutations.

**Table 3:** Clinical and laboratory features of 97 MPNs patients stratified by TET2 status.

	TET2-positive MPN (*n* = 7)	TET2-negative MPN (*n* = 90)	*P* value
Diagnosis			
MPN-U (*n* = 8)	3 (37.5%)	5/8 (62.5%)	
ET (*n* = 55)	2 (3.6%)	53 (96.4%)	0.005
PV (*n* = 25)	2 (8.0%)	23 (92%)	
IMF (*n* = 9)	0	9	
Median age, years (range)	71 (43–76)	65.5 (25–89)	0.57
Follow-up months, median (range)	38.6 (13.3–130)	54.2 (4.3–195.7)	0.38
Hb, g/100 mL (range)	16.1 (13.3–19.9)	15.2 (11.1–21.5)	0.22
WBC × 10^9^/L (range)	6.86 (5.4–12.4)	8.56 (1.7–19.6)	0.3
Monocytes × 10^9^/L (range)	0.3 (0.1–1.15)	0.45 (0.02–0.9)	0.45
Plt × 10^9^/L (range)	476 (134–849)	629 (149–1750)	0.02
JAK 2 V617F *n* (%)	6/7 (85.7)	66/90 (73.3)	0.78
JAK 2 V617F burden median % (range)	24 (5–65)	25 (5–90)	0.99
EEC *n* (%)	4/7 (57.1)	71/90 (78.8)	0.39
CFU-MK *n* (%)	1/7 (14.2)	57/90 (63.3)	0.01
CD34 + SP × 10^9^/L (range)	0.021 (0.0103–0.273)	0.03 (0.0015–0.61)	0.89
CD34 + SP (%) (range)	0.03 (0.02–0.35)	0.035 (0.0015–0.56)	0.43
Bone marrow cellularity % (range)	50 (30–80)	50 (20–98)	0.96
Single cytogenetic abnormality (*n*)	0/7	5/90 (5.5)	0.49
Complex karyotype (*n*)	0/7	1/90 (1.1)	
Trisomy (*n*)	0/7	1/90 (1.1)	
Pruritus (*n*)	2/7	21/90	0.88
Palpable splenomegaly (*n*)	2/7	37/90	0.8
Splenic square cm^2^ (range)	40 (31–105)	42 (25–200)	0.45
Arterial thromboses (*n*)	1/7	21/90	0.43
Venous thromboses (*n*)	2/7	7/90	
Spontaneous haemorrhage (*n*)	*0/7 *	*2/90 *	*0.58 *
Posttraumatic haemorrhage (*n*)	*0/7 *	*2/90 *	
Cytoreductive treatment (*n*)	*7/7 *	*86/90 *	*0.66 *

**Table 4:** Thrombosis in the whole cohort stratified by TET2 mutational status.

	TET2-positive MPN (*n* = 7)	TET2-negative MPN (*n* = 90)	*P* value
Arterial thrombosis			
Ischemic heart syndrome	1/7	13/90	ns
Ischemic stroke	0/7	4/90	

Venous thrombosis		
DVT	2/7	2/90
Portal thrombosis	0/7	3/90	ns
BCS	0/7	1/90	

## References

[1] Tefferi A, Vainchenker W (2011). Myeloproliferative neoplasms: molecular pathophysiology, essential clinical understanding, and treatment strategies. *Journal of Clinical Oncology*.

[2] Abdel-Wahab O, Mullally A, Hedvat C (2009). Genetic characterization of TET1, TET2, and TET3 alterations in myeloid malignancies. *Blood*.

[3] Delhommeau F, Dupont S, Della Valle V (2009). Mutation in TET2 in myeloid cancers. *New England Journal of Medicine *.

[4] Couronné L, Lippert E, Andrieux J (2010). Analyses of TET2 mutations in post-myeloproliferative neoplasm acute myeloid leukemias. *Leukemia*.

[5] Langemeijer SMC, Kuiper RP, Berends M (2009). Acquired mutations in TET2 are common in myelodysplastic syndromes. *Nature Genetics*.

[6] Tefferi A, Pardanani A, Lim KH (2009). TET2 mutations and their clinical correlates in polycythemia vera, essential thrombocythemia and myelofibrosis. *Leukemia*.

[7] Tefferi A, Levine RL, Lim KH (2009). Frequent TET2 mutations in systemic mastocytosis: clinical, KITD816V and FIP1L1-PDGFRA correlates. *Leukemia*.

[8] Tefferi A, Lim KH, Abdel-Wahab O (2009). Detection of mutant TET2 in myeloid malignancies other than myeloproliferative neoplasms: CMML, MDS, MDS/MPN and AML. *Leukemia*.

[9] Abdel-Wahab O (2011). Genetics of the myeloproliferative neoplasms. *Current Opinion in Hematology*.

[10] Tefferi A, Vardiman JW (2008). Classification and diagnosis of myeloproliferative neoplasms: the 2008 World Health Organization criteria and point-of-care diagnostic algorithms. *Leukemia*.

[11] Patriarca A, Pompetti F, Malizia R (2010). Is the absence of JAK2V617F mutation a risk factor for bleeding in essential thrombocythemia? An analysis of 106 patients. *Blood Transfusion*.

[12] Nguyen-Khac F, Lesty C, Eclache V (2010). Chromosomal abnormalities in transformed Ph-negative myeloproliferative neoplasms are associated to the transformation subtype and independent of JAK2 and the TET2 mutations. *Genes Chromosomes and Cancer*.

[13] Saint-Martin C, Leroy G, Delhommeau F (2009). Analysis of the Ten-Eleven Translocation 2 (TET2) gene in familial myeloproliferative neoplasms. *Blood*.

[14] Kosmider O, Gelsi-Boyer V, Ciudad M (2009). TET2 gene mutation is a frequent and adverse event in chronic myelomonocytic leukemia. *Haematologica*.

[15] Kohlmann A, Klein HU, Weissmann S (2011). The Interlaboratory Robustness of Next-generation sequencing (IRON) study: a deep sequencing investigation of TET2, CBL and KRAS mutations by an international consortium involving 10 laboratories. *Leukemia*.

[16] Voso MT, Fabiani E, Piciocchi A (2011). Role of BCL2L10 methylation and TET2 mutations in higher risk myelodysplastic syndromes treated with 5-Azacytidine. *Leukemia*.

[17] Ko M, Huang Y, Jankowska AM (2010). Impaired hydroxylation of 5-methylcytosine in myeloid cancers with mutant TET2. *Nature*.

[18] Pollyea DA, Raval A, Kusler B, Gotlib JR, Alizadeh AA, Mitchell BS (2011). Impact of TET2 mutations on mRNA expression and clinical outcomes in MDS patients treated with DNA methyltransferase inhibitors. *Journal of Hematology & Oncology*.

[19] Szpurka H, Jankowska AM, Makishima H (2010). Spectrum of mutations in RARS-T patients includes TET2 and ASXL1 mutations. *Leukemia Research*.

[20] Jankowska AM, Szpurka H, Tiu RV (2009). Loss of heterozygosity 4q24 and TET2 mutations associated with myelodysplastic/myeloproliferative neoplasms. *Blood*.

